# Livelihood Resilience or Policy Attraction? Factors Determining Households’ Willingness to Participate in Rural Tourism in Western China

**DOI:** 10.3390/ijerph19127224

**Published:** 2022-06-13

**Authors:** Peiying Dang, Linjing Ren, Jie Li

**Affiliations:** 1 School of Public Policy and Administration, Xi’an Jiaotong University, Xi’an 710049, China; peiying8011@stu.xjtu.edu.cn; 2 School of Public Policy and Administration, Northwestern Polytechnical University, Xi’an 710072, China; gonewithwind163@yeah.net

**Keywords:** rural tourism, tourism participation willingness, livelihood resilience, poverty alleviation policy

## Abstract

Rural tourism in developing countries has been regarded as a means for rural development, environment conservation and poverty alleviation. This study aims to examine the determining factors for rural households’ willingness to participate in rural tourism in western areas under the context of China’s rural revitalization strategy. Based on survey data from 22 tourism poverty alleviation villages located in the poor areas of western China, we characterize our results by stating that rural households’ livelihood resilience “push” on their willingness to participate, and that poverty alleviation policy perception and involvement “pull” on it. Among distinct livelihood adaptive strategy groups, i.e., farming-oriented households, migratory-oriented households and tourism-participating households, this study also revealed that buffer capacity was a significant driving force for the three types of household’s willingness. Positive poverty alleviation policy perception could attract migratory households to return to their hometowns to start tourism businesses; however, better self-organizing capacity decreased their willingness. In addition, both self-organization and learning capacity and positive policy perception and involvement encouraged tourism-participating households to engage in tourism activities continually. Finally, some practical implications and recommendations for further research are also discussed.

## 1. Introduction

Over the past 30 years, rural tourism has gradually been considered as a powerful driving force for creating local job opportunities, alleviating absolute poverty, and promoting rural sustainable development in China. However, the tourism participation rate of rural households in poverty-stricken areas has remained low for a long time [1,2]. At the end of 2017, China’s President Xi Jinping first proposed to implement the “Rural Revitalization Strategy” in the report of the 19th National Congress of the Communist Party of China. In 2018, the “Guidance on Promoting the Sustainable Development of Rural Tourism” issued by the national Ministry of Culture and Tourism and 17 other departments of China, emphasized that rural tourism should “take rural residents as the main beneficiary, respect farmers’ willingness, focus on their participatory process, and further increase government’s support and assistance” in order to stimulate tourism employment. It means that rural tourism has been recognized as a feasible approach in subsequent rural revitalization strategy.

As a brand-new industrial poverty alleviation model, rural tourism plays a vital economic role, especially in impoverished areas with abundant tourism resources. However, in the context of China’s rural revitalization implementation, both the internal and external environments that affect rural households’ participation in tourism have gradually changed, particularly in poor areas. First, until recently, China’s rural tourism had reached a level of scale where it needed to be upgraded, which still faces challenges in the form of a single development mode, low level of industrial integration and bottlenecks in family income increases [3]. Second, China’s rural population has been quite stratified due to its recent rapid urbanization. With large numbers of young rural laborers entering cities to seek employment opportunities, rural tourism has faced the problem of a lack of workers to take over [4]. Those laborers who stayed in rural communities are mostly middle-aged and elderly groups, and the operation risk and unstable benefits of tourism employment [3], and being marginalized due to limited assets or capabilities for some poorer ones [5], may also decrease their willingness to participate in tourism. Third, during the targeted poverty alleviation (TPA) period that began in 2013, abundant policy assistance and action plans related to rural tourism have also brought new opportunities for households and tourism industry development [6]. Given these new opportunities and challenges, it is necessary and worthy to explore the rural households’ willingness to participate in rural tourism, which determines how to sustain local residents’ livelihood and upgrade the rural tourism industry in China’s rural revitalization stage.

Many existing studies have focused on the willingness of households to participate in rural tourism [4,7,8,9,10,11,12,13]. Some scholars assessed the perceived impacts of rural tourism on residents’ willingness to participate in rural tourism by using the motivation, opportunity and ability (MOA) model [14] or social representation theory [4], which is mainly from an individual perception perspective. Another strand of most previous studies has focused on the role of livelihood assets in the behavioral choice of tourism participation with analysis based on the sustainable livelihood framework. This framework, which includes five types of livelihood capital, i.e., human, physical, social, financial and natural capital, holds that those with better livelihood capital endowments are more inclined to participate in tourism [8,15,16,17,18]. However, one fact that these studies had ignored is that the capital approach tends to focus on the livelihood choice based only on present conditions and is not forward-looking. In practice, the acquisition and conversion of livelihood capital is a dynamic and changing process that is easily affected by an external and changeable environment [19]. In addition, rural tourism exploitation can be a “double-edged sword” for destination communities. It not only can have major positive impacts on local residents [20], but also can seriously disturb local livelihood systems in its initial stages. For instance, Biggs (2011) [21] found that some farmland expropriated for tourism purposes could change traditional lifestyles and pressure rural ecological environments. Faced with these adverse conditions, farmers’ livelihoods must respond and undergo a positive or negative adaptation process through which the “feasible ability” to cope with shocks can gradually change over time [19]. However, the question of how these changes affect willingness to participate in tourism remains unanswered.

In addition, a new research trend has emerged that focuses on the government’s role and the influence of policy in pushing residents to participate in rural tourism [22,23]. These studies have emphasized that without enough supportive policies, the locals in most cases can only enjoy a marginal share of the benefits from tourism development [24] and also face participation barriers. Additionally, many scholars have focused on the willingness of all residents in rural areas and have not subdivided the populations into relevant groups. Given the household differentiation mentioned above, this does not seem to be an effective solution to identify the ideal subjects that rural tourism should cover and how to consolidate the achievements of poverty reduction through rural tourism during the TPA period in rural China.

In an effort to make up for this, we introduce the “livelihood resilience” approach to describe the “ability” generated from the dynamic adaptive process of rural tourism exploitation. Livelihood resilience research has recently emerged to address issues of how households can potentially adapt to environmental changes [25]. It is a time-bound assessment of the state of a system that can be varied in response to new circumstances. Livelihood resilience can be understood as a “capacity” in accordance with the connotation of resilience, holding that it refers to the ability of a family to respond and adapt to socio-economic and environmental changes by restoring itself from disturbances, learning from changes, and restructuring life and production [26]. Based on the impact of tourism exploitation on the destination community, we aim to use the concept of livelihood resilience to identify whether rural residents could change their ability insufficiency and show increased enthusiasm for tourism participation.

The objective of this study is to explore whether rural households are willing to participate in rural tourism under the internal drive of livelihood resilience (“push” factors) and incentive of tourism poverty alleviation policies (“pull” factors) under the context of China’s rural revitalization strategy. With regard to the specific participants and beneficiaries of rural tourism, patterns of local household participation include involvement in community tourism planning and operations and benefit distribution [27]. This study focuses primarily on participation in tourism operation activities and employment in tourism enterprises, restaurants, and other supporting businesses. The potential contributions to the existing literature are as follows. First, compared with the livelihood capital assets approach, we introduce a “resilience” perspective to capture the capability mechanism of rural households that recovered from tourism exploitation and its possible effects on tourism participation willingness. Second, from the perspective of household perception and involvement, we examine whether poverty alleviation policies can become an attraction for households to participate in rural tourism. Third, we also subdivide the sample households into three types considering the possible differentiation of their willingness at the rural revitalization stage, which has been ignored by previous research.

## 2. Literature Review and Research Hypotheses

### 2.1. Livelihood Resilience and Willingness to Participate in Tourism

As a powerful analytical tool in the domain of sustainability research [26], resilience often describes the capacity of a system or a group to absorb shocks, adapt, or respond, while still maintaining positive function [28,29]. Moreover, it emphasizes the capacity for renewal, re-organization, and development [28]. Resilience was originally applied in a natural environment context but has since been applied to social ecospheres while retaining a similar interpretation [30]. More recently, resilience has been extended from a regional scale to a family and rural households scale as well [26,31,32].

Previous literature emphasizes that the livelihood assets are the dominant factors in the choice of livelihood according to the SL framework [33]. Although this framework offers many useful insights, it also has a number of limitations including that, for example, only examining the impact of the family’s livelihood capital stock on livelihood choices at a specific point of time, and paying insufficient attention to the dynamism progress of family assets changes [19]. Unlike the livelihood assets approach, livelihood resilience refers to the ability of a subject to maintain and increase assets, to self-organize, and to learn to cope with disturbances and stresses [30]. The emphasis of livelihood resilience is on how individuals proactively respond to a changing environment and exert subjective initiative to organize and utilize internal and external resources to achieve better living conditions [26]. In this way, livelihood resilience reflects the individual’s dynamic capacity across temporal and spatial scales that can affect the behavioral intentions of households. Nyamwanza [34] also emphasized that livelihood resilience was also a self-adaptive iterative process including conversions of multiple stable states into each other. Similar to resilience itself, many studies on the livelihood resilience of rural households have shifted from focusing on issues affected by the natural environment [35] to human activities [26].

Based on the analysis, this study indicates that livelihood resilience is embedded in different livelihood activities, and how the livelihood resilience initiates the willingness of tourism participation can be considered into two stages. First, at the early stage of tourism exploitation, the sudden intervention of tourism exploitation shocked locals’ livelihood environment, production and lifestyles [21]. In this process, locals absorbed the positive elements from the shock and then formed different adaptive strategies. Some chose to participate in tourism activities by virtue of their families’ capital endowment advantages, while others still engaged in local agriculture or migrating work, including among them those passively excluded from tourism due to limited assets, lack of labor or social networks [5]. In other words, the households’ present livelihood activities, such as farming, migrant working and existing tourism participation, may be adaptive strategies when confronted with the disturbance of tourism exploitation in its initial stage [36]. Second, based on these present livelihood activities, households may generate different livelihood resilience considering that the sense of livelihood resilience depends on how well a livelihood functions, on a family’s livelihood assets accumulation and the capability to utilize accessible resources, on actors’ capacity for learning, and on distinct livelihood conditions [30,37]. Moreover, livelihood resilience is itself dynamic and changeable and not always in a positive or favorable manner. For example, On the one hand, those who have engaged in tourism inevitably undergo seasonal employment, operational risk, and fluctuations in tourist flow that can result in a poor adaption for tourism development, and this may become a possible concern for subsequent livelihood decisions. On the other hand, when those residents who have not engaged in tourism have accumulated appropriate resources that initial tourism operations required, they may choose to learn the necessary knowledge and skill related to tourism, or to rely on external relations to adapt to a new focus on tourism, and their willingness to participate in tourism may therefore become strengthened. With strong livelihood resilience, households could have the appropriate ability to resist risks and cross the capital “threshold” of tourism participation. Based on the analysis above, we propose the following hypothesis:

**Hypothesis** **1** **(H1).** *The stronger the livelihood resilience, the greater the probability that farmers are willing to participate in rural tourism*.

At present, there are no standardized tools for assessments and quantitative measures of livelihood resilience [32]. Here, we adopt the “3C” dimensions of resilience: buffer capacity, self-organization capacity and learning capacity that have already been widely adopted by many researchers at a micro level [26,31].

#### 2.1.1. Buffer Capacity and Willingness to Participate in Tourism

Buffer capacity refers to a system or an individual’s ability to cushion adverse changes and utilize opportunities to achieve better livelihood outcomes [30]. From a household’s perspective, buffer capacity represents the ability to utilize its own livelihood capital to deal with external disturbances caused by increased tourism exploitation and other factors; it is specifically manifested in the maintenance and accumulation of family capital assets [26].

Many studies have identified that human, financial, physical, and natural capital have a significant influence on the willingness to participate in tourism [8,15]. Bello [16] and Francis, et al. [38] found that inadequate financial funds, insufficient income, and lower-than-average distribution of tourism benefits significantly influenced the willingness to start tourism activities. The strong buffer capacity is conducive for households to breaking through capital barriers, reducing risk expectations, and enhancing their capacity to cope with adverse environments. Hence, we propose the following additional hypothesis:

**Hypothesis** **1a** **(H1a).** *The stronger the buffer capacity, the stronger the willingness of households to participate in rural tourism*.

#### 2.1.2. Self-Organization Capacity and Willingness to Participate in Tourism

Self-organization capacity refers to a household’s capacity to shape flexible, changeable, and cooperative networks, as well as its ability to integrate into local social, economic, and institutional environments [21,30]. This could be reflected by the social network, organization participation, or dependence on a household’s own resources [26,30]. With self-organization, households can obtain favorable development opportunities and employ external resources to achieve their livelihood goals.

Researchers have found that social norms, power relations [39], relationships of trust and reciprocity, and collective norms [15] are critical to rural residents’ participation in tourism. Tourism development has been found to have broken their original social networking structures. This is because several new stakeholders enter the community, including tourism developers, foreign business operators, and tourism company employees, which complicates the existing social network of tourism communities. Those who have an active self-organizing capacity, however, can quickly integrate into the new atmosphere and seek favorable opportunities related to tourism, thereby avoiding becoming marginalized. In addition, as Avila-Foucat and Rodriguez-Robayo [17] and Bhatta et al. [40] have indicated, engaging in certain community organizations and frequently becoming involved in community activities are two key factors that help to determine whether a household participates in tourism. Chinese rural households have a deep-rooted tradition of collective action. When joining a given community organization, they can share information with other community members, unite to bargain with tourism developers, and then decrease the cost of each individual’s action. Based on this, we propose the following additional hypothesis:

**Hypothesis** **1b** **(H1b).** *The stronger the self-organization capacity, the stronger the willingness of households to participate in rural tourism*.

#### 2.1.3. The Learning Capacity and Willingness to Participate in Tourism

The learning capacity refers to the ability to acquire knowledge and skill through imitation, transference, improvement, and creation to update an individual’s past experiences and knowledge [26,30]. At the individual level, learning ability can be acquired through perceptions as well as through theoretical knowledge [30]. For poverty reduction motivation, local governments often offer several systematic training items related to tourism business operation and service skills in an attempt to enhance residents’ tourism participation capacity, and several studies have found that knowledge and perception have positive effects on the tourism participation of indigenous people [41]. Reversely, inadequate tourism knowledge and skills can be constraints for tourism participation [16,42].

In addition, rural households can improve their learning capacity through practical methods such as social interactions and work experience [30]. Specifically, they may imitate and learn from others to gain practical experience or skills [26]. For instance, those who have yet to participate in tourism may directly learn from current tourism participants or obtain experience from public media, which itself could increase the willingness to pursue tourism livelihood activities, especially for those who are quick learners. With a strong learning ability, households can quickly master tourism operation skills and enhance their confidence in participating in tourism activities. Based on this, we propose the following additional hypothesis:

**Hypothesis** **1c** **(H1c).** *The stronger the learning capacity, the stronger the willingness of households to participate in rural tourism*.

### 2.2. Poverty Alleviation Policy and Willingness to Participate in Tourism

It is widely recognized that China has adopted a “government-led” strategy for its tourism development [43]. Within China’s TPA period, the implementation of the government-led poverty alleviation tourism policy has played a central role in lifting poor people out of poverty and diversifying rural income sources in impoverished areas [44]. In 2016, rural tourism benefited 6.72 million farmers [45], and according to 2019 data from the Ministry of Culture and Tourism, national rural tourism poverty alleviation in their monitored rural areas accounted for 30.6% of the poor’s employment [46].

Rural tourism is an essential component of China’s poverty alleviation by industrial development. As an anti-poverty means, it aims to support the poor households who have the ability to work and possess the related skills to engage in rural tourism as a livelihood and to help them solve employment difficulties. In the implementation of TPA, China’s governments at various levels mainly take the local’s resource endowment or skills as the foundation, and the community tourism employment capacity and household demand as the guidance to supply accurate poverty alleviation policy and assistance measures. For example, those poor households who want to pursue tourism operation activities but lack of funds could receive industrial investment subsidies or micro-finance assistance; families who have participated in agritainment could also be covered by such a tax exemption policy. Besides, some inclusive measures such as tourism skills training, agritainment star rating, and online tourism marketing aim to enhance the internal driving force of poor regions and populations [47].

The role of the government and poverty alleviation policy in rural residents’ participation in tourism and poverty reduction has become an area of academic interest in recent tourism research [22,23]. Scholars have argued that policy orientation can be the institutional guarantee for residents’ tourism participation, can help to lift the poor out of poverty, can relieve the poor’s marginalization by other stakeholders and increase benefits from tourism development [48], resulting in a positive perception of tourism by locals [44]. This perception can also have a significant and positive impact on the tourism community’s overall participation in tourism-related industries [49]. Residents’ perceptions of tourism policies show to some extent their concern and benefits for government intervention [44]. In addition, during the most recent period of targeted poverty alleviation in China, some households have obtained direct assistance under the policy and benefited from rural industrial development. This type of involvement may have provided its beneficiaries with a better understanding of tourism poverty alleviation policies and assistance measures, thereby improving the attractiveness of tourism for rural households. Based on this, we propose the following hypotheses:

**Hypothesis** **2a** **(H2a).** *The better the perception about tourism poverty alleviation policies, the more households are willing to participate in rural tourism*.

**Hypothesis** **2b** **(H2b).** *The deeper involvement of poverty alleviation assistance, the more households are willing to participate in rural tourism*.

**Figure 1:**
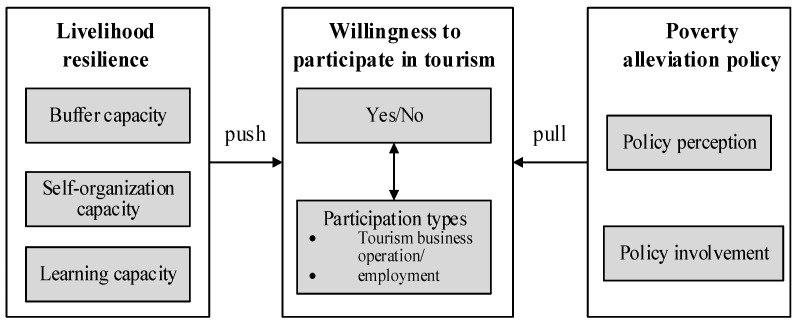
The analytical framework for the willingness to participate in tourism of rural households based on livelihood resilience and poverty alleviation policies.

To test our hypotheses, we built an analytical framework, as shown in Figure 1. In the next sections of this study, we introduce study areas and data and conduct empirical tests. Finally, we discuss results and present our conclusions.

## 3. Methodology

### 3.1. Study Sites and Data Sources

The survey area of this study is located in the national Qinba Mountains’ poverty-stricken areas in western China. The main area of interest is located in the southern region of Shaanxi Province, which has abundant and diverse natural and ecological resources. Here, rural tourism has already become an effective tool for local employment, poverty alleviation and rural revitalization and received significant attention from governments at all levels [50].

However, the Qinba area remains one of the 14 contiguous areas of dire poverty and includes most of the poor population in western China. As shown in Table 1, the sample areas we surveyed included four cities of Shaanxi Province: Baoji, Ankang, Hanzhong, and Shangluo, cities which rural tourism exploited earlier and have since formed distinct characteristics. The surveyed villages are close to the 3A level and above primary scenic spots that local households have benefited from tourism for several years. Furthermore, these rural tourism spots have all been designated as typical demonstration sites for poverty alleviation by rural tourism administrators at the national or provincial level. In addition, the survey period we selected is also the peak tourist season. Many respondents, including some migratory workers that go outside to do part-time jobs, are observed to engage in tourism. Therefore, several years of tourism development, the appropriate time to investigate, and representative poverty alleviation policy interventions all make the surveyed villages good representatives for investigating the livelihood resilience that the locals have recovered from tourism exploitation, and for exploring the correlations between locals’ resilience, poverty reduction policy perception and involvement and willingness to participate in tourism.

From June to July in 2017, we collected data in the above four cities. The sampling process was as follows. First, we took the first group of rural tourism-based poverty alleviated villages in Shaanxi Province as the sampling frame and used stratified random sampling to select 22 villages in four cities. Second, we used a convenience sampling method and structured questionnaires to interview the household heads aged 18–65 or their spouses who stayed at home at the time of the investigation and chose them randomly.

Moreover, several measures were taken to ensure data quality and reliability, including prior surveys conducted in 2017 from 12 to 14 June in Ankang, on 15 and 16 June in Shangluo, and from 15 to 29 June in Baoji and Hanzhong, to determine the suitability of the surveyed villages and to correct inappropriate items on the questionnaire. In-depth interviews were conducted for some key respondents, such as leaders of tourism bureaus, agricultural bureaus, poverty alleviation bureaus, and village directors and cadres, to acquire details about the village tourism development. Pre-training for investigators, a face-to-face interview with each one, and on-site guidance for the formal survey were also included. Finally, we distributed 875 questionnaires to the sample sites, and 841 valid questionnaires were returned, for an effective rate of 97.68%.

### 3.2. Variables

#### 3.2.1. The Dependent Variable

The dependent variable is the willingness to participate in rural tourism, which refers to whether local residents are willing to participate in a tourism business operation or to be employed by tourism enterprises or in other tourism-related jobs. When asked about their willingness to participate in rural tourism in the future, the respondents in surveyed communities could answer either “yes” or “no”, so this is a binary variable.

#### 3.2.2. The Independent Variables

(1) Measurement of Livelihood Resilience

Drawn on the resilience dimensionalities proposed by Speranza, Wiesmann and Rist [30] and other studies [26,31,32,37,51], as well as the information on household characteristics in the study areas, we established a set of indicators and variables to measure buffer capacity, self-organization capacity, and learning capacity, as shown in Table 2.

(2) Policy Perception and Involvement of Rural Households

From the rural households’ perspective, the effects of poverty alleviation policies on tourism participation exists in two aspects: policy perception and policy involvement (Table 2). Policy perception refers to the understanding of tourism poverty alleviation policies and measures, and the perception of tourism exploitation policy affects households’ employment opportunities. Policy involvement refers to the amount of poverty alleviation policy assistance, and projects that the households received, such as industrial investment subsidies, microfinance, tax exemptions, and assistance projects, including tourism skills training, agritainment star rating, anti-poverty projects through returns on asset investments, and poverty alleviation resettlement subsidies. In addition, China’s land transfer policy is also an important policy intervention that can affect the land use of farmers and tourism development. Hence, we also include the variable “whether the farmland transferred for tourism development” to reflect how the land use policy affects farmers’ attitudes towards rural tourism (Table 2).

Due to the indicators above having different types and standards, this study utilizes, firstly, the min-max normalization method to transform the original variable value into a standard value so as to achieve dimensional consistency. After that, all of the variable values fall in the range of [0, 1]. The equal weight method is then adopted to calculate the weights of variables, given that each variable is of equal importance within the three dimensions of livelihood resilience and poverty alleviation policy. For example, when we calculate the buffer capacity index (*BC*), we use the formula as follows:

$$BC=\sum X_{i}*W_{i}$$

where $X_{i}$ represents the range standardization value of the sub-indicators of buffer capacity (Table 2), and $W_{i}$ represents the weight of the *i* indicator. In the same way, we can calculate the self-organization capacity index, learning capacity index, policy perception and policy involvement variables.

#### 3.2.3. Rural Households Types

Based on the indicators of the proportion of tourism employment in the family and the proportion of different sources of family income (standardized values), we use K-means cluster analysis to cluster the samples and combine the clustering results with standardized data to correct for deviations. To accomplish this, we divide households into three types. The farming-oriented type has family livelihood activities that are dominated by agroforestry production, though some have a small amount of local temporary non-agricultural jobs in nearby areas, and this accounted for 36.32% of the total sample households. The migratory-oriented type accounted for 26.52% of the total sample and it refers to “the major workforce working outside in most times in a year and a little agroforestry during the farming seasons” livelihood type. Here, most of the young family members or household heads go out for non-farm employment, while some elderly members maintain agricultural and forestry production. The tourism-participating type is characterized by tourism-related livelihood activities and accounted for 37.16 % of the total sample. The forms of tourism participation include agritainment, commodity retailing, or stable employment in tourism enterprises, but some family members take care of agroforestry or go out for part-time jobs during the off-season.

### 3.3. The Binary Logistic Model

Considering the distribution characteristics of the dependent variable, we employ a binary logistic model to analyze the factors that affect households’ willingness to participate in rural tourism. The regressions were conducted in both the total samples and sub-samples of the above three household types using the following Equation:

$$\mathrm{Logistic}\left(Y\right)=\beta_{0}+\beta_{i}X_{i}+\mu_{i}$$

where *Y* refers to the households’ willingness to participate in rural tourism, $X_{i}$ refers to the indicators of livelihood resilience including buffer capacity, self-organization capacity, learning capacity, and poverty alleviation policy perception and involvement, and $\mu_{i}$ refers to the residual terms of the model.

## 4. Results

### 4.1. Descriptive Statistics of Variables

**Figure 2:**
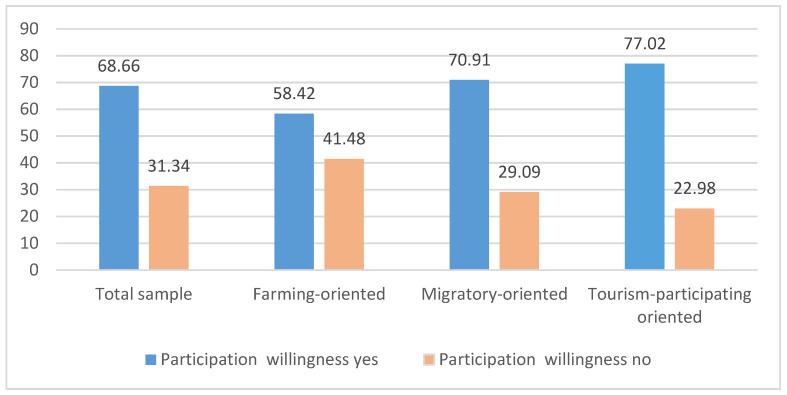
Distribution of rural households’ willingness to participate in rural tourism in surveyed areas (%).

The distribution of rural households’ willingness to participate in tourism in the survey areas is shown in Figure 2. Overall, 68.66% of the sample residents were willing to participate in rural tourism. For different types of households, the proportions that were willing were 58.42% (farming-oriented type), 70.91% (migratory-oriented type), and 77.02% (tourism-participating type), respectively. Evidently, most of the households who have engaged in tourism activities tended to continue to participate in tourism in the future, while those who primarily operate agricultural enterprises showed less willingness to adjust to tourism livelihood activities although they have temporal-spatial convenience compared with migratory types of households. Surprisingly, though, nearly 23% of the tourism participants still showed an unwillingness to engage in tourism in the future.

### 4.2. Econometric Model Results

#### 4.2.1. Determinants of Willingness to Participate in Tourism

Table 3 presents the estimates from our binary logistic regression of livelihood resilience, policy perception and involvement in households’ willingness to participate in rural tourism. Though not previously mentioned, we also controlled for the influence of regional variables (city-level) in all models to better study the impact of the primary independent variables on the dependent variables. As observed in Table 3, the stronger the buffer capacity, self-organization capacity and learning capacity, the stronger the willingness of households to participate in rural tourism. Given these results, we find support for H1, H1a, H1b, and H1c. Also, we find that poverty alleviation policy perception and involvement have a significant influence on rural residents’ willingness. That is, a better perception of tourism poverty reduction policies and a higher amount of poverty alleviation assistance can have an increased likelihood that farmers are willing to participate in rural tourism. Hence, H2a and H2b are supported.

With the four models in Table 4, we examine the effect of sub-indicators of livelihood resilience and poverty alleviation policy on the willingness, respectively. Model 1 primarily studies the effect of variables of buffer capacity on the dependent variable. Specifically, if rural residents have a larger housing area, better housing structure, better loan opportunities, and higher per capita income, they prefer to engage in tourism livelihood activities. Sufficient human capital, such as family labor, also has a significant positive impact at the 1% level. In the same vein, the positive impact of self-organization capacity sub-indicators on the dependent variable is presented in Model 2, where we see that social support networks and participation in village public affairs play significant roles in enhancing households’ willingness to participate. However, the impact of whether to become involved in cooperatives and the number of cadre relatives are not significant. According to Model 3, learning ability significantly positively affects willingness to participate in tourism through skills, training, and education level of the household head rather than through time of working outside for family members. Finally, Model 4 demonstrates the significant impact of poverty reduction policy sub-indicators on willingness. The understanding of tourism poverty alleviation policies significantly strengthens rural households’ intentions to choose a tourism livelihood; the perception of tourism job opportunities and the number of poverty alleviation projects or items have positive effects as well. Additionally, if households transfer their farmland to tourism enterprises or cooperatives for tourism development, their willingness to participate also increases.

#### 4.2.2. Differences in the Influencing Factors of Willingness to Participate in Tourism among Different Household Types

Table 5 shows the estimates of influencing factors of willingness to participate in tourism among different types of households, and Table 6 demonstrates the regression results of sub-indicators. From the two tables we can see that the determinant factors of willingness have differed between farming-oriented, migratory-oriented and tourism-participating households in livelihood resilience and poverty reduction policy as well as their sub-indicators.

According to Table 5 and Table 6, buffer capacity reflected by housing area, loan availability and labor availability have significantly positive effects for farming-oriented households, but self-organization capacity and the capacity for learning are not statistically significant. We also see that the more positive policy perception the households have, the more they are willing to participate in rural tourism. In detail, understanding of tourism poverty alleviation policy and the perception of job opportunities are significantly and positively related to willingness; meanwhile, policy support items and land transfer related to tourism have positive impacts on the willingness but they are not significant (Table 5), in accordance with the effect of the policy involvement variables in Table 6. Overall, buffer capacity and policy perception may be the critical factors for farming-oriented households to shift to tourism livelihood activities.

As reflected in the migratory-oriented sub-sample, buffer capacity dimension has a positive influence on the tourism participation willingness at the 5% significance level. In other words, housing structure and loan availability are the buffer capacity factors that most affect the dependence variable. As for the self-organization capacity dimension, the interesting finding is that the stronger the self-organization capacity, the less is the willingness. For specific sub-indicators, the number of cadre relatives has a significant negative impact on the migrant workers’ willingness to participate in tourism. However, in terms of learning capacity, skills and working outside time did not have a significant effect. In addition, for the policy perception, when migrant households perceive that tourism could create more employment opportunities for their communities, they are more likely to return to their hometowns to participate in tourism activities. These results indicate that buffer capacity, self-organization capacity, and poverty alleviation policy perception jointly affect the willingness to participate in tourism.

For the tourism-participating sub-sample, Table 5 shows that both the three dimensions of livelihood resilience and poverty alleviation policy play significant roles in the willingness. As shown in Table 6, if households have more products and tools, better loan availability and social support networks, or if they join in cooperatives or they are often involved in village public affairs, their willingness increases. Similarly, willingness also strengthens when family members have received training and the household head has a higher level of education. Moreover, we do find that the sub-indicators of policy perception and involvement positively increase the willingness. These results indicate that buffer capacity, self-organization and learning capacity, as well as poverty alleviation policy perception and involvement are the key factors for current tourism participants to keep participating in tourism activities in the future.

## 5. Discussion

### 5.1. Livelihood Resilience Acts as a “Push”

Compared to the existing literature, this study explores the determining factors of willingness to participate in tourism with the background of China's rural revitalization strategy. We contend that rural households’ livelihood resilience acts as a “push” in enhancing the households’ willingness to participate in rural tourism, not only by the maintenance and accumulation of family assets but also by the improvements of households’ self-organization and learning capabilities. Rural tourism is a labor-intensive industry that has the potential to create jobs for locals. However, at the early stage of tourism development, those who have limited family assets have less freedom to engage in tourism activities [5,8], but this is not set in stone. Under the context of Chinese traditional culture, rural households often prefer saving and storing resources, especially in poor rural areas; part of a family’s income is often saved for building new houses, cash storage, investing in reproduction, and for coping with other livelihood risks [26]. These preferences generate the buffer capacity represented by the stock and increment of households’ capital [26], which could exert a driving force on the willingness to participate in tourism.

Certain social networks and organizational foundations can significantly decrease the vulnerability of households to diverse environments and strengthen their capacity for organizing external resources to improve livelihood resilience [26,32,37], and this extensive benign social connection can enhance farmers’ probability to participate in tourism [15,39]. Furthermore, our empirical results indicate that the more frequent the participation in village public affairs, the more households tend to be involved in rural tourism. In China’s rural communities, village committees can help organize households and relate to each other. Residents who often positively participate in village public affairs may know more about community tourism development details such as the newest policies and rules, market opportunities, employment information, and other stakeholder relations, and may express their opinions in a formal way that helps to decrease marginalization and to promote the benefits of tourism participation. However, the empirical models also show that whether to become involved in cooperatives had no significant impact on households’ willingness, possibly because these cooperatives mostly operate agroforestry and breeding and are not closely related to the tourism development in our survey areas.

Effective community participation in tourism requires that community members are necessarily skilled, trained, and relatively educated [16,38,40]. With a strong learning capacity, households can put acquired skills and knowledge into practice, which in turn can affect their livelihood activity choice. This study also finds that a higher learning capacity increases willingness to participate in tourism. One way this may occur is that households can directly imitate and learn from each other and gain more experience in tourist-related activities. In addition to this, some formal training from local governments or enterprises can supply some practical techniques related to tourism like cooking, serving, and various operating businesses that can remedy the inability of households and increase their willingness to participate in rural tourism.

The results demonstrate that the most important factor that determines farming-oriented households’ willingness to participate in rural tourism is buffer capacity. In practice, this type of household often has a low-income level as a result of the low efficiency and output of agroforestry in western China. However, some of these households are less likely to go out for nonfarm jobs due to the older age of household heads and their high dependency ratios. As a result, buffer capacity reflected by maintained and accumulated wealth is often lower than other types of households, which then become the constraint on engaging in tourism. That is, if this capacity breaks the “threshold” for participating in tourism, then the willingness will increase for farming-oriented households.

Migratory-oriented households usually have stronger buffer capacity than farming-oriented ones, owing to their relatively higher nonfarm income. At present, however, due to economic recession and the “working-outside economy” depression in China, rural households are having more trouble with finding stable jobs in urban settings than they did previously [53]. Furthermore, the high cost of urbanization prevents rural laborers from integrating into the city. Actually, when nonfarm income levels are similar to farm income levels, rural households tend to pursue job opportunities closer to their homes so as to reduce their cost of living and to take care of family members [54]. This study finds that buffer capacity is also the key factor for migrant workers’ willingness to participate in tourism, which is similar to the findings of Wang and Sun’s [12] study indicating that in rural communities where the tourism industry has developed, migrant laborers with better family resource endowments are inclined to return home for tourism employment. Interestingly, households with better self-organizing capacity show less willingness to participate. Obviously, strong social networking is beneficial for households to advance livelihood recovery and acquire stable nonfarm jobs outside the community [55], as family bonds closely with cadre relatives and neighbors. According to our interview, local families with at least one to two members who have stable non-agricultural employment are more willing to continue engaging in their current livelihood activities than to participate in rural tourism due to the relatively higher wages in the city. Associated with this, migrant working time, often representing the greater possibility to accumulate practical experience and enhanced learning capacity, had no significant influence on willingness. The possible reason could be that even if these migratory-oriented households are advantaged in terms of learning capacity, the question of whether they wish to return home to pursue tourism activities is a matter of weighing the pros and cons between urban and rural areas.

In contrast to the other two types of households, the three dimensions of livelihood resilience play active roles in these households’ willingness for tourism-participating households. Not surprisingly, livelihood assets are originally the basis of tourism participants to choose the tourism-related livelihood activities, but then the wealth of families increased and the buffer capacity was further enhanced, thereby becoming the driving force of continuous willingness to engage in tourism. Additionally, according to our interview, those initially engaged in tourism livelihood activities at the early stage of tourism exploitation usually have a better ability to resist tourism operational risks. In particular, favorable loan availability could encourage further investment in households’ tourism operations. However, the transformation of households’ livelihoods from their previous livelihoods to a stable tourism livelihood is a continuous process [56], and the requirements for resilience are also emphasized differently during discrete periods of tourism development. In the early stages, these groups participate in tourism activities by virtue of livelihood capitals, but with the gradual deepening of their participation, their capacity for self-organization and for acquiring knowledge and skills becomes more pivotal. Here, simple home-based inns can no longer satisfy the increasing expectations of tourists and must shift toward a composite model that integrates leisure, health, and entertainment, all of which require a higher level of knowledge, skill, and operating capability. This result is similar to the previous study by Xue and Kerstetter [56], showing that rural households’ self-organization capacity and learning capacity can play a key role in promoting their willingness to participate in tourism at the post-development era of rural tourism. Specially, those with strong learning abilities and who have new skills training related to tourism can better adapt to the transformation of tourism development and maintain their willingness to participate in tourism.

### 5.2. Poverty Alleviation Policy Acts as a “Pull”

Many studies have quantitatively analyzed the effects of the government’s promotion of tourism development in China’s poor areas through certain policies, rules, and regulations, including the introduction of external investment to develop tourism and expand local employment [57] and loans, subsidies, or skill training provisions targeted to the poor [1] to improve their ability to participate in tourism.

The empirical results of this study support the idea that deep and detailed perception and involvement of the poverty alleviation policies can act as a “pull” to lift the willingness of rural households to participate in tourism. Poverty alleviation policy implementation in China enables them to have a deep understanding of the goals, steps, and the pro-poor nature of poverty alleviation policies. In particular, a positive perception of tourism employment opportunities may act as an attraction and demonstration for migrant laborers returning to their hometowns to start a business, which may be conducive to attracting capable labors dedicated to rural revitalization [12]. More importantly, we find that the farming-oriented households who have positive perceptions of government policies are more eager to become involved in tourism; and for tourism participants, poverty reduction policy assistance they have received may lead them to view such policies positively and to therefore become more willing to participate in rural tourism.

As China maintains a “strong and active national government”, especially with regard to efforts aimed at fighting poverty [26], rural households’ livelihood activities may indeed be influenced and guided by these efforts. At present, the Chinese government has proposed that all of the targeted poverty reduction policies continue to implement and establish special institutions and policies to guarantee long-acting support so as to consolidate their existing achievements of targeted poverty reduction in the rural revitalization stage. Based on our findings, this proposal may also stabilize rural households’ willingness to continue to participate in tourism activities.

## 6. Conclusions

This study aims to explore the determinant factors of rural households’ willingness to participate in rural tourism in impoverished areas in the context of China’s rural revitalization strategy. It also contributes significant value for other developing countries to develop rural tourism, expand community participation and achieve comprehensive poverty reduction. First, the empirical findings indicate that livelihood resilience can act as a positive “push” force to rural households’ willingness to participate in tourism. In particular, family housing conditions, labor, loan opportunities, and per capita income can enhance the buffer capacity of rural households and thereby increase their willingness. Social support networks that consist of relatives and friends, and frequent village public affairs participation are also beneficial to households’ self-organizing abilities and further increase willingness. For learning capacity, skills, training, and education levels of the household head have significant positive effects on willingness as well. Second, rural households with a better understanding of tourism poverty reduction policy and a more positive perception of tourism employment in their communities as well as more policy support items targeted to families have a more active willingness to engage in tourism. Third, these influencing factors varied among different household types. Farming-oriented households have the lowest willingness, followed by the migratory-oriented households, and tourism-participating ones have the highest. Buffer capacity is a significant driving force for the three types of household willingness. Positive poverty alleviation policy perception could attract migratory households to return to their hometowns to start tourism businesses; however, better self-organizing capacity decreased their willingness. For tourism-participating households, strong self-organizing and learning capacity, as well as positive policy perception and involvement, can incentivize them to continue to be willing to participate in tourism.

Several limitations of this study should be mentioned. First, in addition to the determinants from a household perspective as examined in this study, several external factors such as modes of tourism development, stakeholder relations in tourism communities and the type and attraction of scenic spots can also be addressed in further research. Second, resilience attributes with dynamics, time-bound, and temporal and spatial changes. However, we only chose the specific investigation time after a period of the tourism exploitation nearby communities, and use the cross-sectional survey to examine livelihood resilience in different livelihood adaptive strategies, which possibly cannot accurately capture the process of variation of livelihood resilience. Third, livelihood resilience and poverty alleviation factors might not be independent but combine together to play a role in enhancing willingness. For example, those with stronger resilience may have a more positive policy perception, or perhaps they have a better understanding of tourism poverty alleviation policies and assistance measures. Owing to the limitation of the research method and article length, this needs to be considered in further analysis.

Nevertheless, this study contributes to China’s ongoing efforts to encourage community tourism participation and identify the role of poverty reduction policy in stimulating household enthusiasm for rural tourism in poverty-stricken areas. Based on the research results, the following aspects should be promoted: a long-term livelihood resilience capacity building mechanism is essential and should be guaranteed in rural tourism communities, paying attention to the changes in participation willingness caused by livelihood resilience level improvement, such as livelihood capitals accumulation and skills acquisition. Moreover, household willingness differentiation should be noted, especially for those who have not yet participated in tourism but have strong willingness, and specific targeted policy support assistance to remedy their “short board” of endogenous resilience is indispensable. Lastly, it is necessary to keep poverty reduction policy continuity and publicity and policy implementation in accordance with household needs; more importantly, further investment should be strengthened to promote the sustainable development of rural tourism so as to increase the attractiveness of rural tourism employment.

## Figures and Tables

**Table 1:** The distribution of survey areas, sample villages, and scenic spots.

City	County/District	Sample Village	Scenic Spots *	Samples
Ankang	Ningshan	Qili, Xujiacheng	Tongchewan Scenic Area (4A)	144
		Shagou	Qinling Canyon Rafting Area (3A)	46
	Shiquan	Yonghong, Zhongba	Zhongba Grand Canyon (4A)	160
	Hanbin	Qingquan, Guojiahe	Yinghu Scenic Area (4A)	83
Shangluo	Shangnan	Taiziping, Miaotaizi, Hetaoping	Jinsixia Scenic Area (5A)	48
	Danfeng	Wanwan, Dihua	Dihua Ancient Town (4A)	76
Hanzhong	Xixiang	Luozhen, Huilong,	Luojiaba Scenic Area (4A)	40
	Chenggu	Liujiaying, Xiaobei	Juyuan Scenic Area (3A)	41
Baoji	Chencang	Nanyu, Anli, Xinmin	Dashuichuan International Resort (4A)	80
	Mei	Shangwang, Yanjiabao, Honghe	Taibaishan Scenic Area (5A)	123

Note: * The star rating of scenic spots refers to that of the tourist attraction assessed by China’s former National Tourism Administration at the time of our survey.

**Table 2:** Indicators of livelihood resilience, poverty alleviation, policy perception and involvement for rural households.

Dimension	Variables	Definition and Description	Mean (SD)	Literature Citation
Buffer capacity	Housing area	The housing area of rural households per capita (m^2^)	179.667 (165.856)	[30]
	Housing structure	Civil structures = 0.33, brick and wood structures = 0.67, brick-concrete structures = 1.00	0.868 (0.244)	[51]
	Products and tools	The number of fixed assets per capita of families: domestic appliances, tricycle, motorcycle, car, agricultural tools, etc. (pieces)	1.168 (0.810)	[37]
	Loan availability	Whether to borrow from a bank or friends (1 = yes, 0 = no)	0.281 (0.357)	[32]
	Labor availability	The number of household membersbetween 18–65 years old (number)	2.759 (1.376)	[31,32]
	Per capita income	Annual household income per capita (Yuan *) (log)	7.606 (2.312)	[52]
Self-organization capacity	Social support network	The number of households available to offer monetary help when families suffer from large expenses (number)	3.893 (3.995)	[31,51]
	Involvement in Cooperative	Whether to join in farmers’ cooperatives for rural households (1 = yes, 0 = no)	0.098(0.297)	[25,35,51]
	Village public affairs participation	The Participation in public affairs in the village the year before survey: 1 = none, 2 = seldom, 3 = less, 4 = some, 5 = a lot	2.339 (1.259)	[30,35]
	Cadre relatives	The number of relatives who work in village or township cadre relatives or in other public official positions (number)	0.606 (1.640)	[31,51]
The learning capacity	Skill	Whether the family members master a skill: cooking, weaving, tailoring, carpentry, bricklaying, etc. (1 = yes,0 = no)	0.341 (0.474)	[31,35]
	Training	Whether the family members received technology training related to planting, aquaculture, cooking, etc. (1 = yes,0 = no)	0.159 (0.365)	[31]
	Household head education	1 = college and above, 2 = senior high school or technical high school, 3 = junior high school, 4 = primary school, 5 = literacy	3.310 (0.863)	[26,35]
	Working outside time	Time of working outside of family labor the year before survey, characterizing the opportunity to acquire cognitive abilities (months)	8.668 (9.323)	[26]
Policy perception	Understanding for tourism poverty alleviation policy	The understanding for tourism poverty alleviation policies and measures: 1 = very little, 2 = little, 3 = a little, 4 = some, 5 = very much	2.489 (1.068)	questionnaire
	Perception for tourism employment opportunities	Perception of the impact of tourism-type poverty reduction policies on a family’s employment opportunities: 1 = greatly reduced, 2 = reduced, 3 = not changed, 4 = increased, 5 = greatly increased	3.510 (0.640)	questionnaire
Policy involvement	Policy support items	The amount of poverty alleviation policy assistance and projects received by households (number)	0.716 (1.023)	questionnaire
	Land transfer related with tourism	Whether to transfer farmland for tourism development (1 = yes, 0 = no)	0.093 (0.290)	questionnaire

Notes: * 1 USD = 6.6401 CNY in 2016.

**Table 3:** The regression estimates of willingness to participate in tourism for rural households (total sample).

Variables	Coefficient	Std. Err	*Z*-Value
Buffer capacity	0.932 ***	0.156	5.96
Self-organization capacity	0.185 **	0.092	2.01
The learning capacity	0.273 **	0.128	2.14
Policy perception	1.679 ***	0.298	5.64
Policy involvement	0.434 *	0.251	1.73
Region	yes		
Constant	−3.542 ***	0.463	−7.64
LR chi^2^	122.58		
Prob > chi^2^	0.000		
Pseudo R^2^	0.118		

Note: *** *p* < 0.01, ** *p* < 0.05, * *p* < 0.1.

**Table 4:** The regression estimates of willingness to participate in tourism for rural households in sub-indicators (total sample).

Variables	Model1	Model2	Model3	Model4
Housing area	0.001 * (0.001)			
Housing structure	0.750 ** (0.328)			
Products and tools	0.128 (0.116)			
loan availability	0.969 *** (0.242)			
Labor availability	0.189 *** (0.064)			
Per capita income	0.102 *** (0.034)			
social support network		0.080 *** (0.025)		
Involvement in Cooperative		0.169 (0.296)		
Village public affairs participation		0.216 *** (0.067)		
Cadre relatives		0.022 (0.053)		
Skill			0.408 ** (0.171)	
Training			0.604 ** (0.242)	
Household head education			−0.278 *** (0.093)	
Working outside time			0.001(0.008)	
understanding for tourism poverty alleviation policy				0.258 *** (0.082)
Perception of tourism employment opportunities				1.114 *** (0.146)
Policy support items				0.116 * (0.076)
Land transfer related with tourism				0.624 ** (0.309)
Region	yes	yes	yes	yes
Constant	−2.040 ** (0.443)	−0.167 (0.210)	1.273 *** (0.356)	−3.973 *** (0.546)
LR chi^2^	67.81	32.21	29.52	99.10
Prob > chi^2^	0.000	0.000	0.000	0.000
Pseudo R^2^	0.066	0.031	0.029	0.096

Notes: Standard errors are in parentheses; *** *p* < 0.01, ** *p* < 0.05, * *p* < 0.1.

**Table 5:** Differences in the influencing factors of different types of rural households’ willingness to participate in rural tourism.

Variables	Farming-Oriented Type	Migratory-Oriented Type	Tourism-Participating Type
Buffer capacity	1.081 *** (0.265)	0.809 ** (0.365)	0.538 * (0.286)
Self-organization capacity	0.302 (0.304)	−0.612 * (0.360)	0.937 ** (0.418)
The learning capacity	0.263 (0.214)	−0.067 (0.264)	0.518 ** (0.222)
Policy perception	1.956 *** (0.476)	1.953 *** (0.588)	1.178 ** (0.552)
Policy involvement	0.298 (0.390)	0.794 (0.551)	0.274 * (0.153)
Region	yes	yes	yes
Constant	−4.793 *** (0.790)	−2.773 *** (0.971)	−2.260 ** (0.896)
LR chi^2^	66.07	24.22	32.87
Prob > chi^2^	0.000	0.002	0.000
Pseudo R^2^	0.161	0.091	0.099

Notes: Robust standard errors are in parentheses; *** *p* < 0.01, ** *p* < 0.05, * *p* < 0.1.

**Table 6:** Differences in the influencing factors of different types of rural households’ willingness to participate in rural tourism (in sub-indicators).

Variables	Farming Oriented Type	Migratory-Oriented Type	Tourism-Participating Type
Housing area	0.003 * (0.002)	−0.003 (0.002)	0.002 (0.001)
Housing structure	0.311 (0.575)	1.774 ** (0.700)	−0.208 (1.015)
Products and tools	0.239 (0.214)	−0.157 (0.282)	0.386 * (0.230)
loan availability	1.297 *** (0.451)	1.230 ** (0.594)	0.804 * (0.464)
Labor availability	0.215 * (0.118)	0.001 (0.158)	0.023 (0.136)
Per capita income	−0.006 (0.054)	0.091 (0.199)	−0.003 (0.093)
social support network	0.008 (0.035)	−0.062 (0.057)	0.114 ** (0.056)
Involvement in Cooperative	0.180 (0.509)	−0.846 (0.719)	1.480 * (0.873)
Village public affairs participation	0.150 (0.139)	−0.038 (0.166)	0.270 * (0.149)
Cadre relatives	0.159 (0.136)	−0.200 * (0.112)	−0.004 (0.167)
Skill	0.262 (0.330)	0.233 (0.400)	0.493 (0.331)
Training	−0.578 (0.554)	−0.145 (0.757)	2.045 *** (0.690)
Household head education	−0.187 (0.173)	0.001 (0.228)	−0.305 * (0.184)
Working outside time	0.000 (0.018)	−0.030 (0.020)	−0.036 * (0.019)
Understanding for tourism poverty alleviation policy	0.296 * (0.158)	0.301 (0.196)	0.255 (0.180)
Perception of tourism employment opportunities	1.136 *** (0.265)	0.985 *** (0.370)	0.799 *** (0.273)
Policy support items	0.177 (0.218)	0.102 (0.266)	0.554 *** (0.203)
Land transfer related with tourism	0.512 (0.462)	1.139 (0.841)	1.546 * (0.837)
region	yes	yes	yes
Constant	−6.491 *** (1.402)	−4.452 ** (2.071)	−1.783 (1.682)
LR chi^2^	85.62	41.30	57.95
Prob > chi^2^	0.000	0.005	0.000
Pseudo R^2^	0.214	0.158	0.182

Notes: Robust standard errors are in parentheses; *** *p* < 0.01, ** *p* < 0.05, * *p* < 0.1.

## Data Availability

Data available on request due to privacy/ethical restrictions.
